# Supplementary material for: ANKEF1 is a key axonemal component essential for murine sperm motility and male fertility
Source: eLife. 2025 Dec 29;14:RP105321. doi: 10.7554/eLife.105321 (PMC12747526; doi:10.7554/eLife.105321)
Supplement: Figure 4—source data 1. [file elife-105321-fig4-data1.zip › Figure 4_Source data 1/Figure 4-Source data 1.pdf]

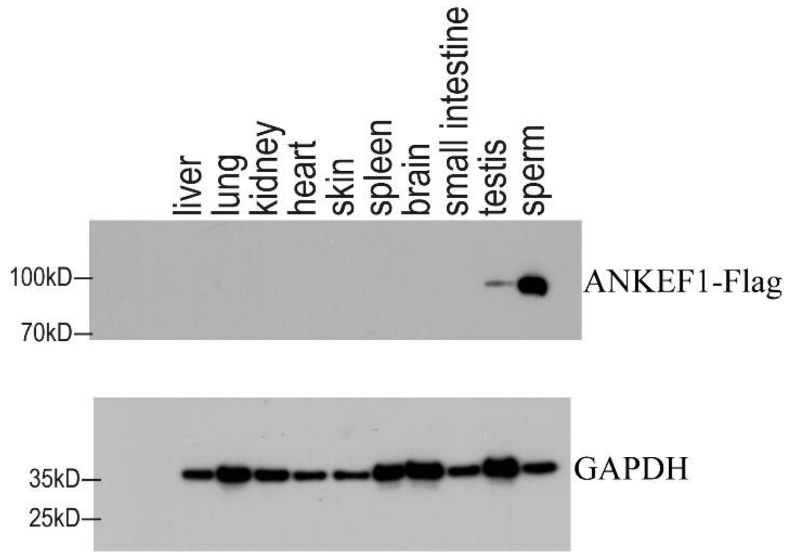

Figure 4, Source Data 1. Original, uncropped western blot membranes corresponding to Figure 4A. The upper membrane was probed with anti-Flag antibody, and the lower membrane with anti-GAPDH antibody. Lanes 1–10 correspond to protein lysates from the following mouse tissues: liver, lung, kidney, heart, skin, spleen, brain, small intestine, testis, and sperm. Pre-stained protein molecular weight markers were used (See Supplementary File 2 for antibody details).
