# Supplementary figures and images for: ANKEF1 is a key axonemal component essential for murine sperm motility and male fertility
Source: eLife. 2025 Dec 29;14:RP105321. doi: 10.7554/eLife.105321 (PMC12747526; doi:10.7554/eLife.105321)

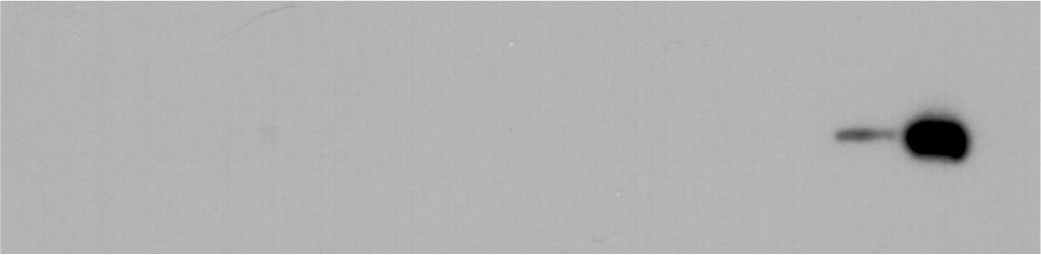

Supplement: Figure 4—source data 2. [file elife-105321-fig4-data2.zip › Figure 4_Source data 2/ANKEF1-Flag.tif]

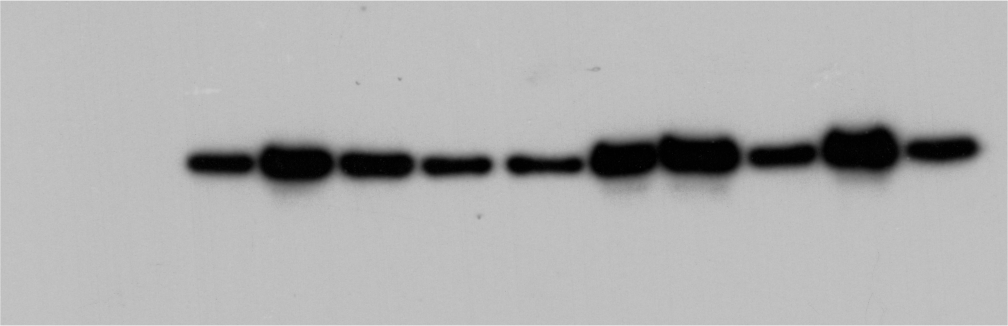

Supplement: Figure 4—source data 2. [file elife-105321-fig4-data2.zip › Figure 4_Source data 2/GAPDH.tif]

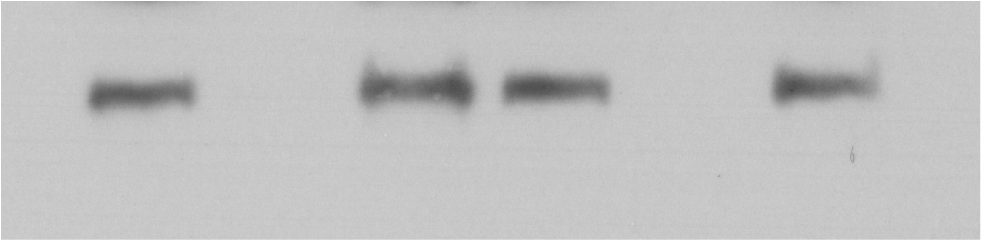

Supplement: Figure 4—source data 4. [file elife-105321-fig4-data4.zip › Figure 4_Source data 4/acetylated-tubulin.tif]

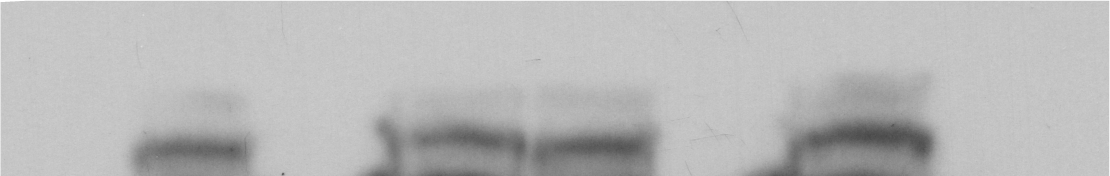

Supplement: Figure 4—source data 4. [file elife-105321-fig4-data4.zip › Figure 4_Source data 4/AKAP4.tif]

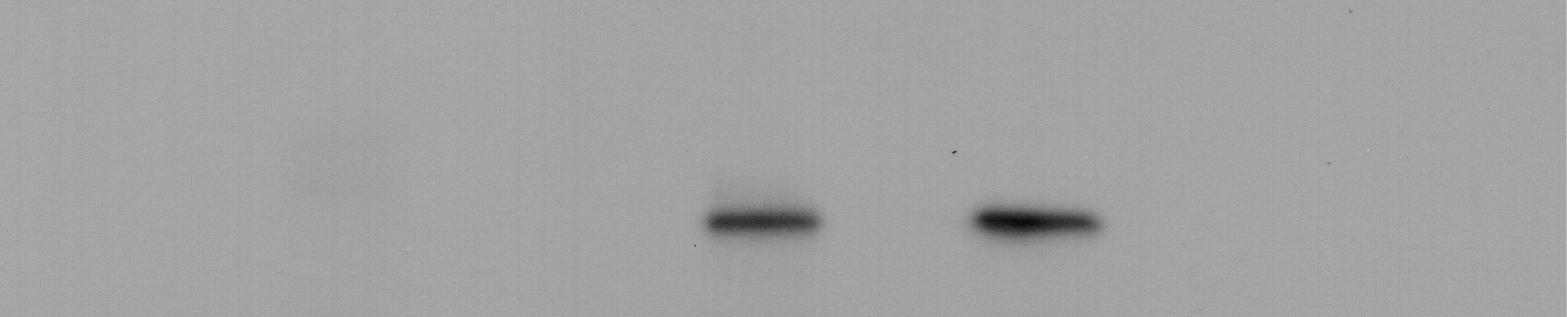

Supplement: Figure 4—source data 4. [file elife-105321-fig4-data4.zip › Figure 4_Source data 4/ANKEF1-Flag.tif]

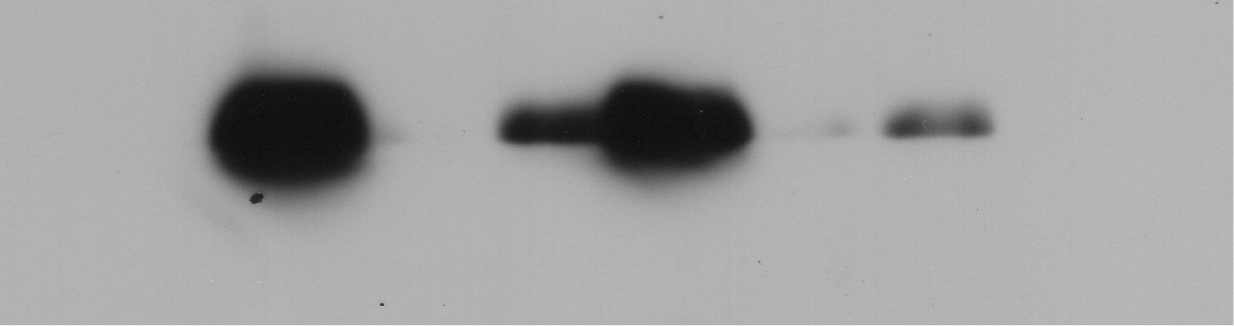

Supplement: Figure 4—source data 4. [file elife-105321-fig4-data4.zip › Figure 4_Source data 4/BASIGIN.tif]

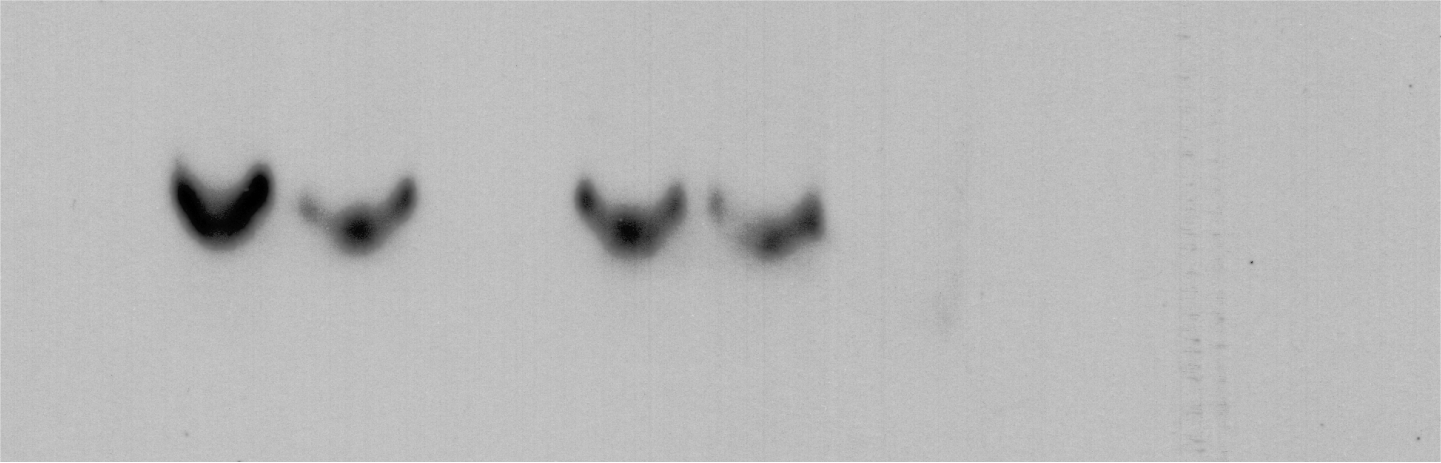

Supplement: Figure 4—source data 4. [file elife-105321-fig4-data4.zip › Figure 4_Source data 4/PRM2.tif]

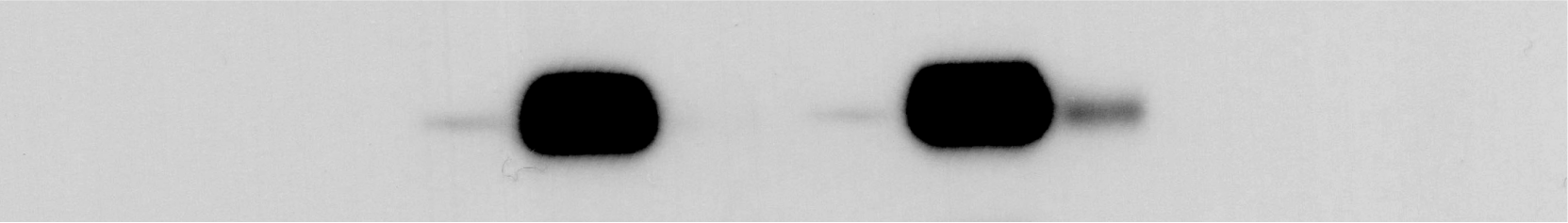

Supplement: Figure 4—source data 6. [file elife-105321-fig4-data6.zip › Figure 4_Source data 6/acetylated-tubulin.tif]

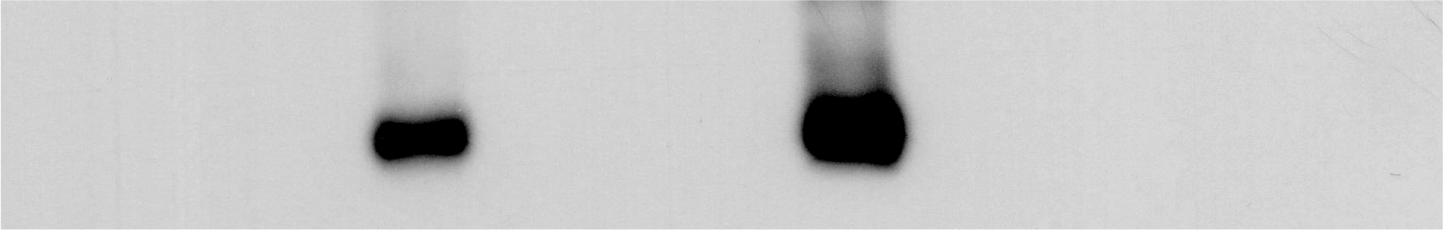

Supplement: Figure 4—source data 6. [file elife-105321-fig4-data6.zip › Figure 4_Source data 6/ANKAP4.tif]

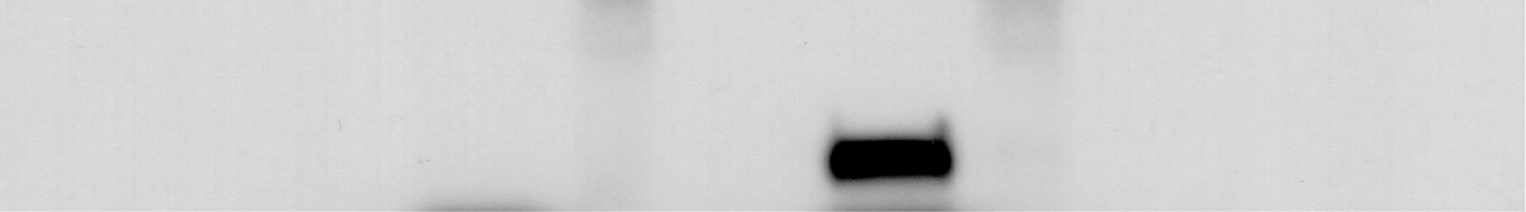

Supplement: Figure 4—source data 6. [file elife-105321-fig4-data6.zip › Figure 4_Source data 6/ANKEF1-Flag.tif]

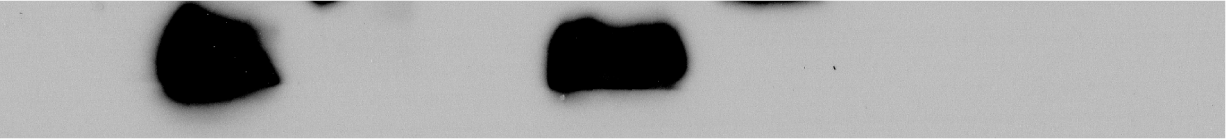

Supplement: Figure 4—source data 6. [file elife-105321-fig4-data6.zip › Figure 4_Source data 6/BASIGIN.tif]

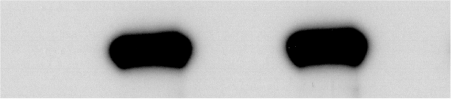

Supplement: Figure 5—source data 2. [file elife-105321-fig5-data2.zip › Figure 5_Source data 2/ANKEF1-Flag-1.tif]

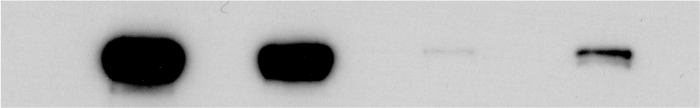

Supplement: Figure 5—source data 2. [file elife-105321-fig5-data2.zip › Figure 5_Source data 2/ANKEF1-Flag.tif]

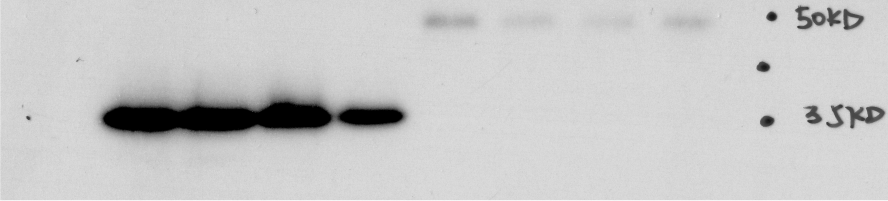

Supplement: Figure 5—source data 2. [file elife-105321-fig5-data2.zip › Figure 5_Source data 2/GAPDH.tif]

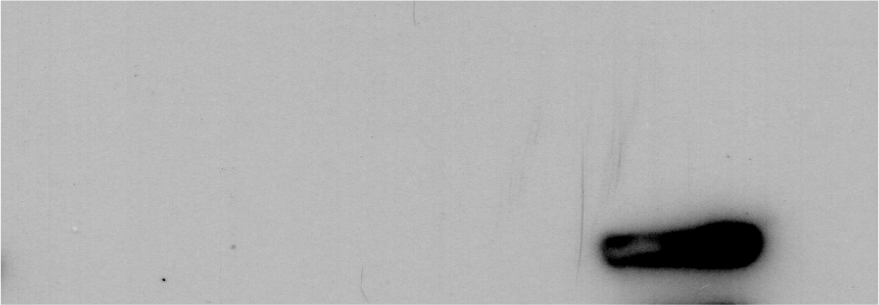

Supplement: Figure 5—source data 2. [file elife-105321-fig5-data2.zip › Figure 5_Source data 2/GAS8-Myc-1.tif]

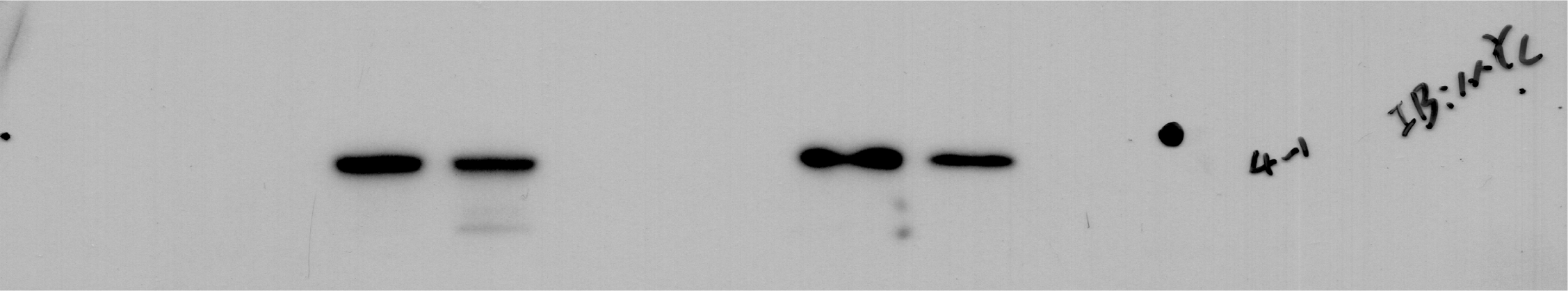

Supplement: Figure 5—source data 2. [file elife-105321-fig5-data2.zip › Figure 5_Source data 2/GAS8-Myc.tif]

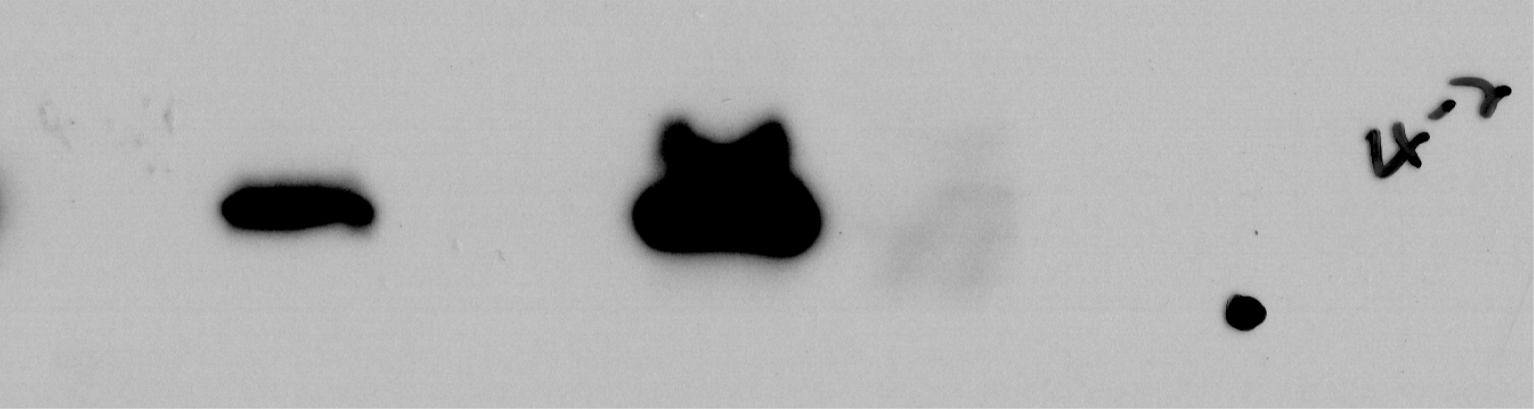

Supplement: Figure 5—source data 4. [file elife-105321-fig5-data4.zip › Figure 5_Source Data 4/ANKEF1-Flag-1.tif]

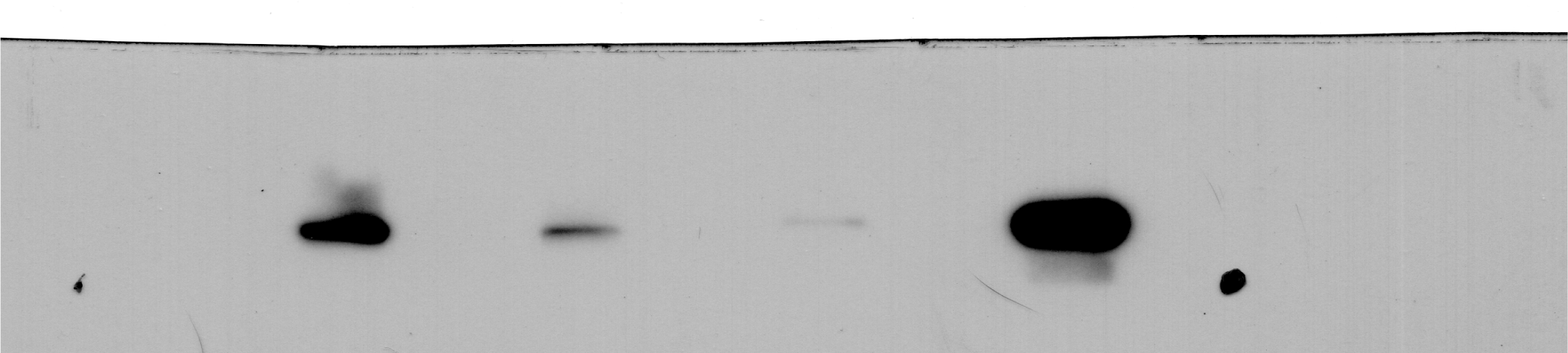

Supplement: Figure 5—source data 4. [file elife-105321-fig5-data4.zip › Figure 5_Source Data 4/ANKEF1-Flag.tif]

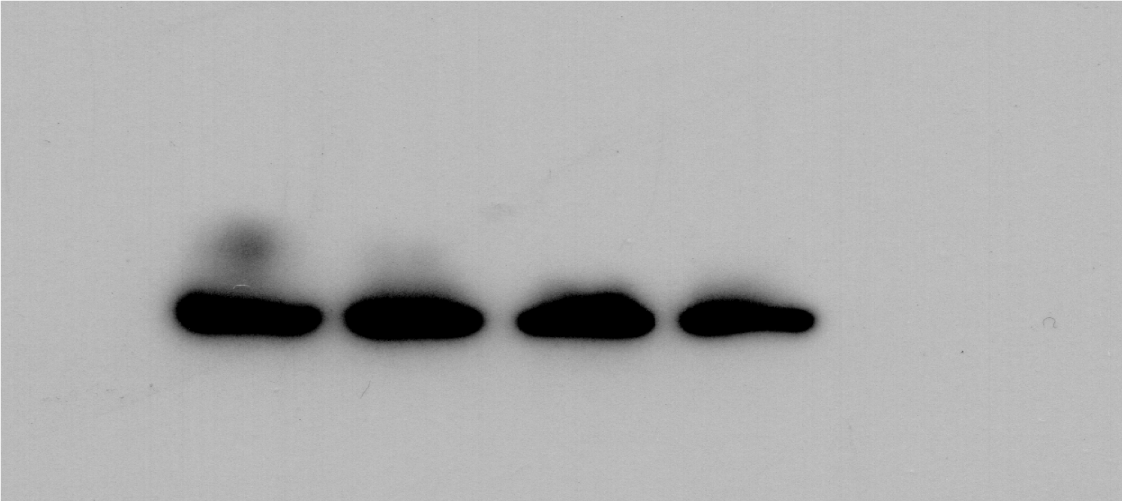

Supplement: Figure 5—source data 4. [file elife-105321-fig5-data4.zip › Figure 5_Source Data 4/GAPDH.tif]

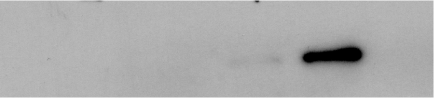

Supplement: Figure 5—source data 4. [file elife-105321-fig5-data4.zip › Figure 5_Source Data 4/TCTE1-Myc-1.tif]

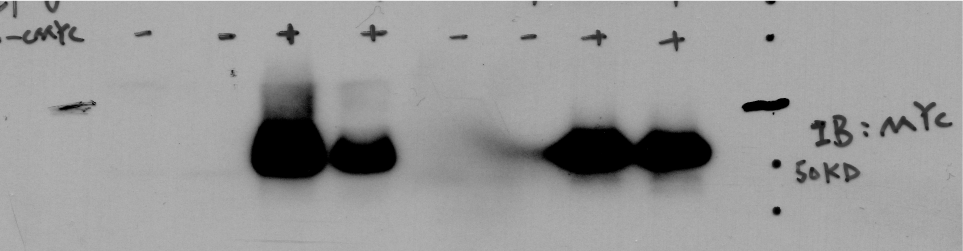

Supplement: Figure 5—source data 4. [file elife-105321-fig5-data4.zip › Figure 5_Source Data 4/TCTE1-Myc.tif]

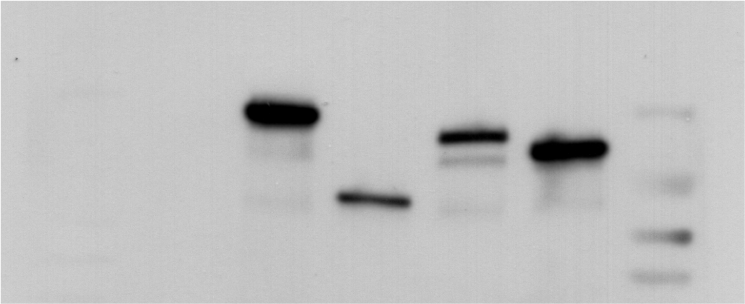

Supplement: Figure 5—source data 6. [file elife-105321-fig5-data6.zip › Figure 5_Source Data 6/ANKEF1-Flag-1.tif]

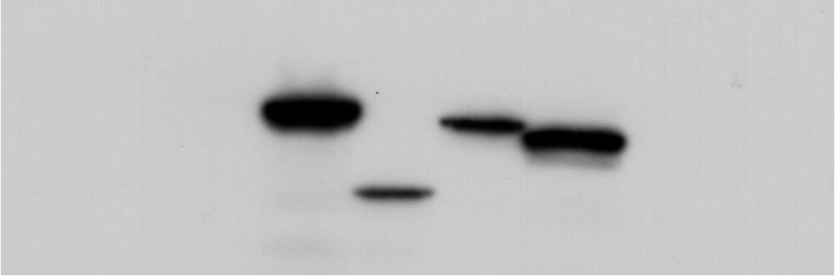

Supplement: Figure 5—source data 6. [file elife-105321-fig5-data6.zip › Figure 5_Source Data 6/ANKEF1-Flag.tif]

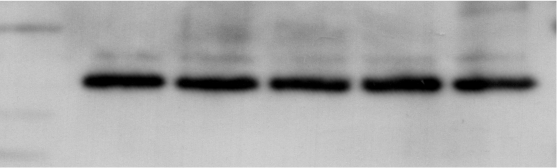

Supplement: Figure 5—source data 6. [file elife-105321-fig5-data6.zip › Figure 5_Source Data 6/GAPDH.tif]

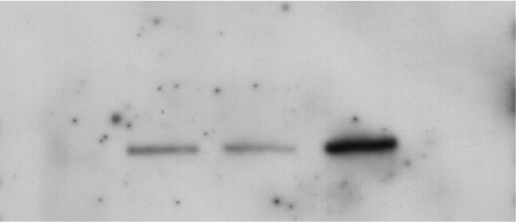

Supplement: Figure 5—source data 6. [file elife-105321-fig5-data6.zip › Figure 5_Source Data 6/TCTE1-Myc-1.tif]

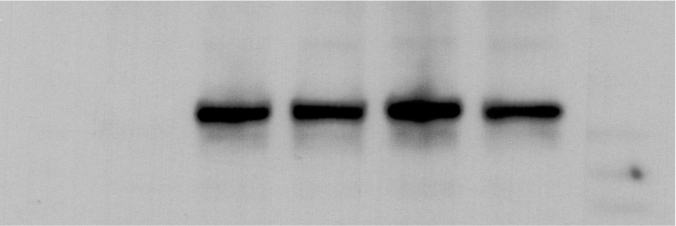

Supplement: Figure 5—source data 6. [file elife-105321-fig5-data6.zip › Figure 5_Source Data 6/TCTE1-Myc.tif]

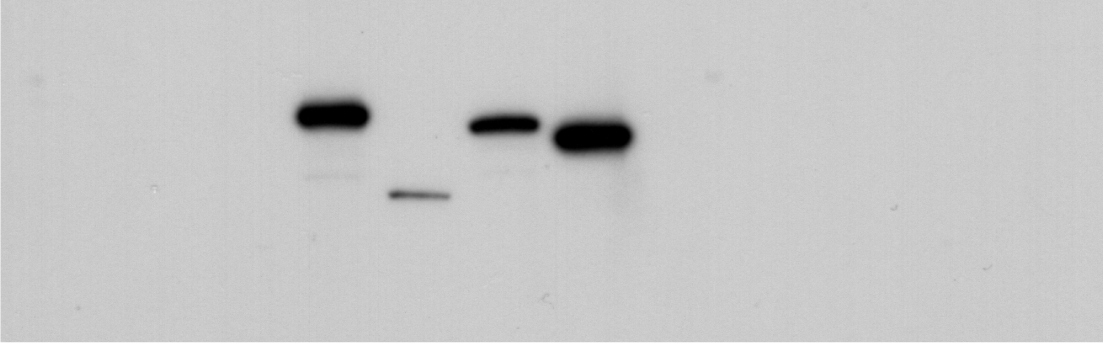

Supplement: Figure 5—source data 8. [file elife-105321-fig5-data8.zip › Figure 5_Source Data 8/ANKEF1-Flag-1.tif]

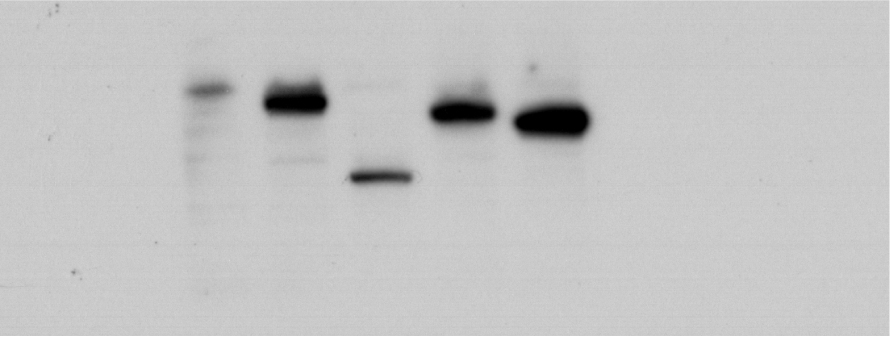

Supplement: Figure 5—source data 8. [file elife-105321-fig5-data8.zip › Figure 5_Source Data 8/ANKEF1-Flag.tif]

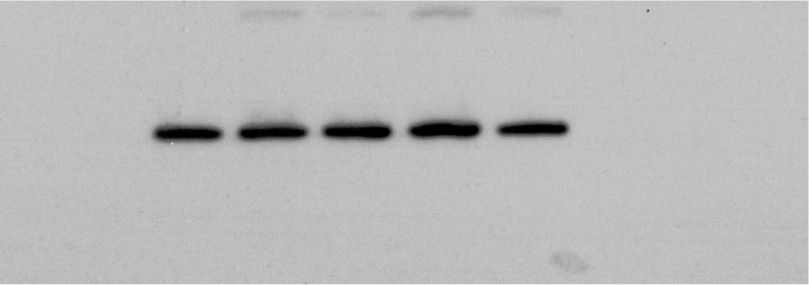

Supplement: Figure 5—source data 8. [file elife-105321-fig5-data8.zip › Figure 5_Source Data 8/GAPDH.tif]

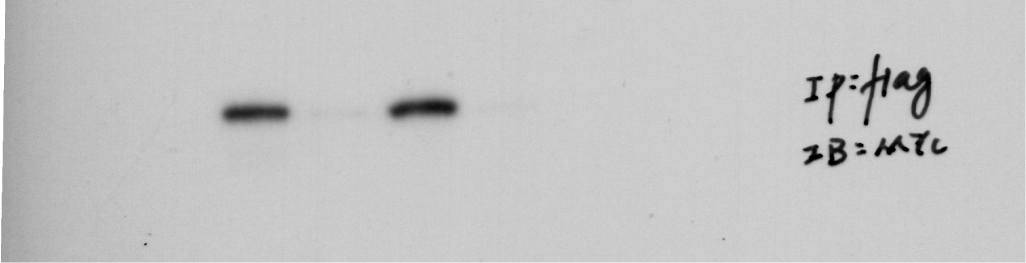

Supplement: Figure 5—source data 8. [file elife-105321-fig5-data8.zip › Figure 5_Source Data 8/GAS8-Myc-1.tif]

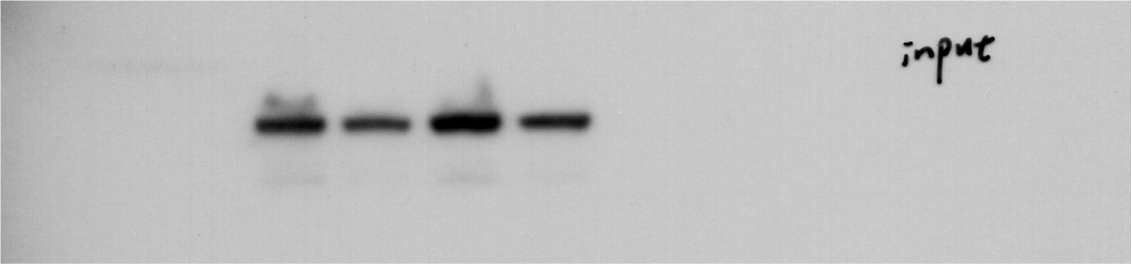

Supplement: Figure 5—source data 8. [file elife-105321-fig5-data8.zip › Figure 5_Source Data 8/GAS8-Myc.tif]

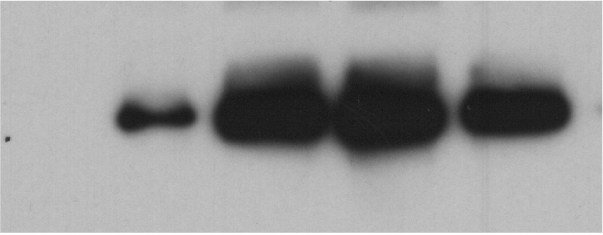

Supplement: Figure 5—source data 10. [file elife-105321-fig5-data10.zip › Figure 5_Source Data 10/ANKEF1-Flag-1.tif]

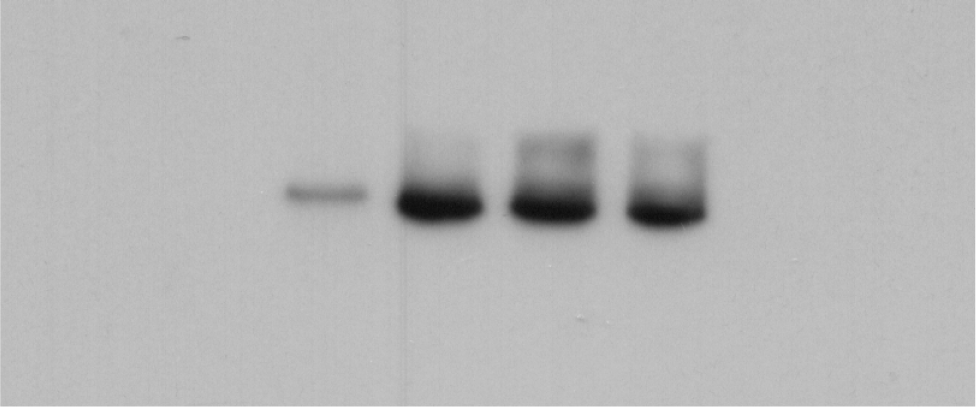

Supplement: Figure 5—source data 10. [file elife-105321-fig5-data10.zip › Figure 5_Source Data 10/ANKEF1-Flag.tif]

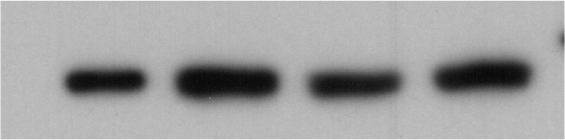

Supplement: Figure 5—source data 10. [file elife-105321-fig5-data10.zip › Figure 5_Source Data 10/GAS8-Myc-1.tif]

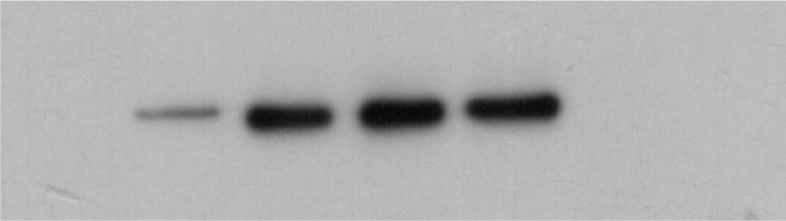

Supplement: Figure 5—source data 10. [file elife-105321-fig5-data10.zip › Figure 5_Source Data 10/GAS8-Myc.tif]

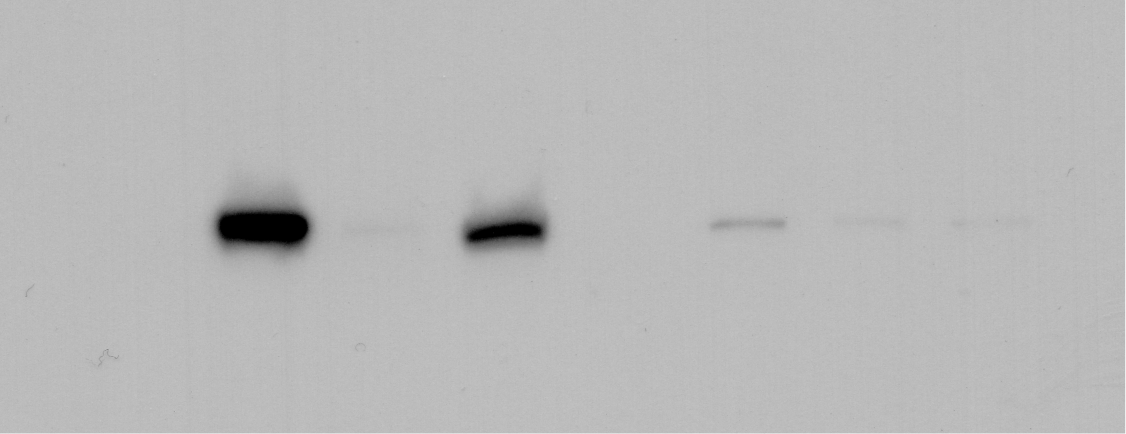

Supplement: Figure 5—figure supplement 1—source data 2. [file elife-105321-fig5-figsupp1-data2.zip › Figure 5-figure supplement 1_Source data 2/ANKEF1-Flag.tif]

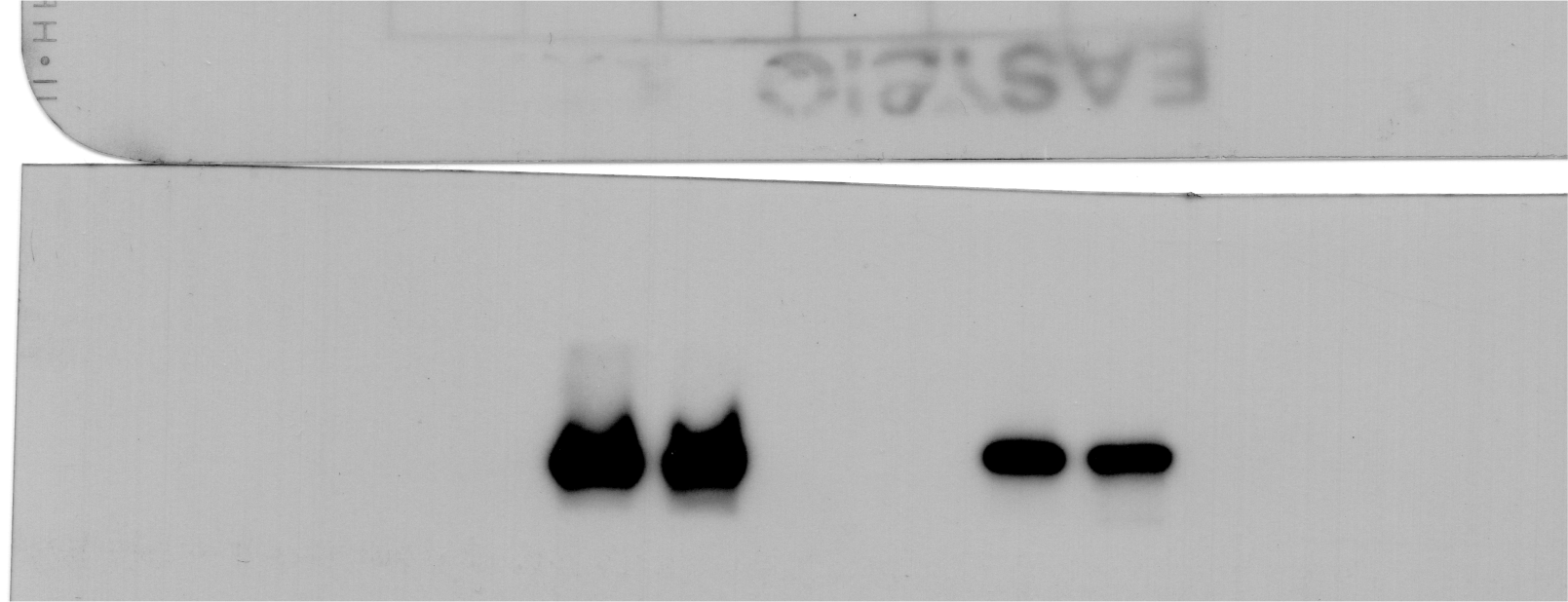

Supplement: Figure 5—figure supplement 1—source data 2. [file elife-105321-fig5-figsupp1-data2.zip › Figure 5-figure supplement 1_Source data 2/DRC1-Myc.tif]

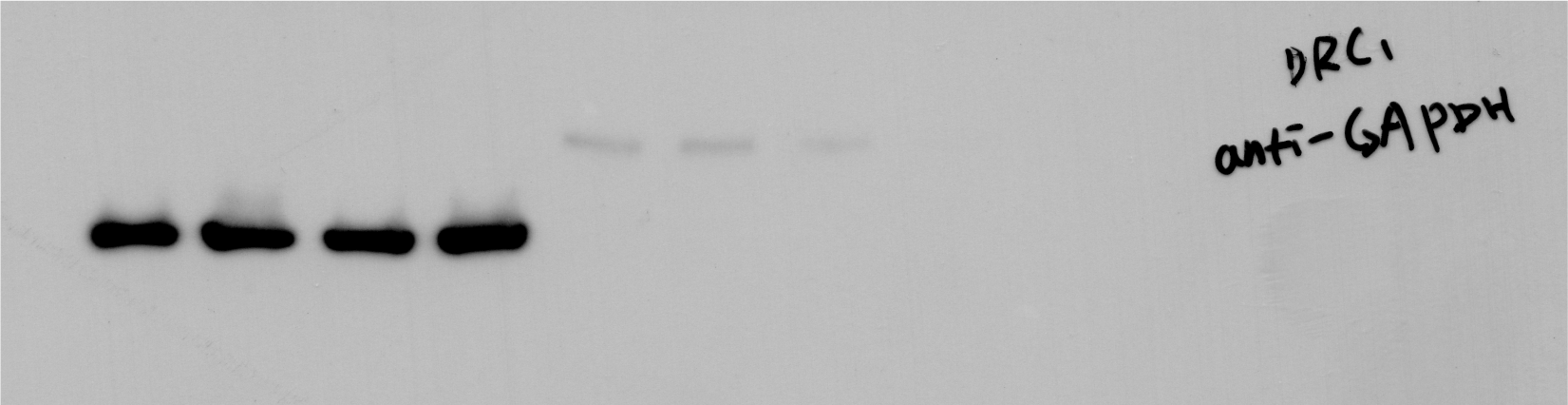

Supplement: Figure 5—figure supplement 1—source data 2. [file elife-105321-fig5-figsupp1-data2.zip › Figure 5-figure supplement 1_Source data 2/GAPDH.tif]

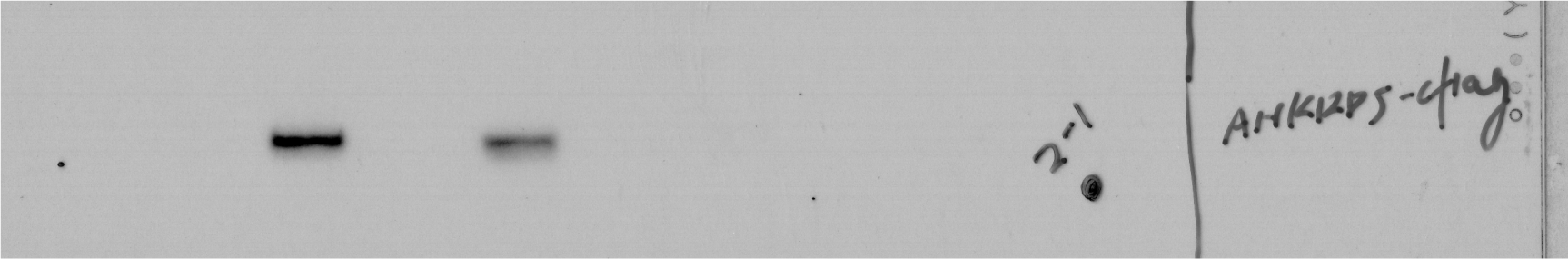

Supplement: Figure 5—figure supplement 1—source data 4. [file elife-105321-fig5-figsupp1-data4.zip › Figure 5-figure supplement 1_Source data 4/ANKEF1-Flag.tif]

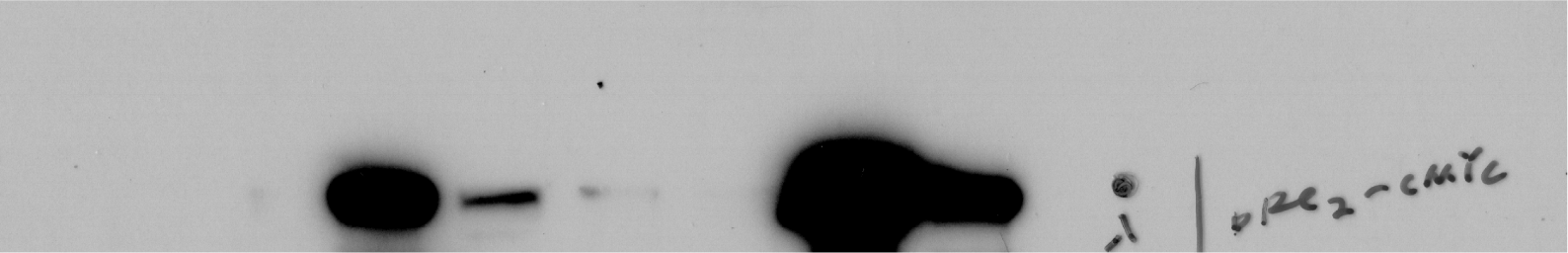

Supplement: Figure 5—figure supplement 1—source data 4. [file elife-105321-fig5-figsupp1-data4.zip › Figure 5-figure supplement 1_Source data 4/DRC2-Myc.tif]

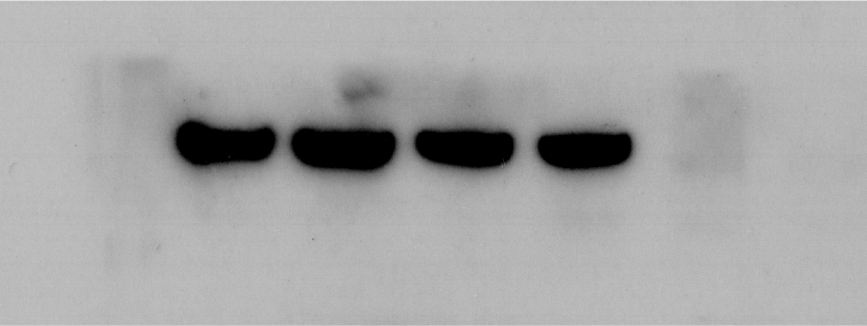

Supplement: Figure 5—figure supplement 1—source data 4. [file elife-105321-fig5-figsupp1-data4.zip › Figure 5-figure supplement 1_Source data 4/GAPDH.tif]

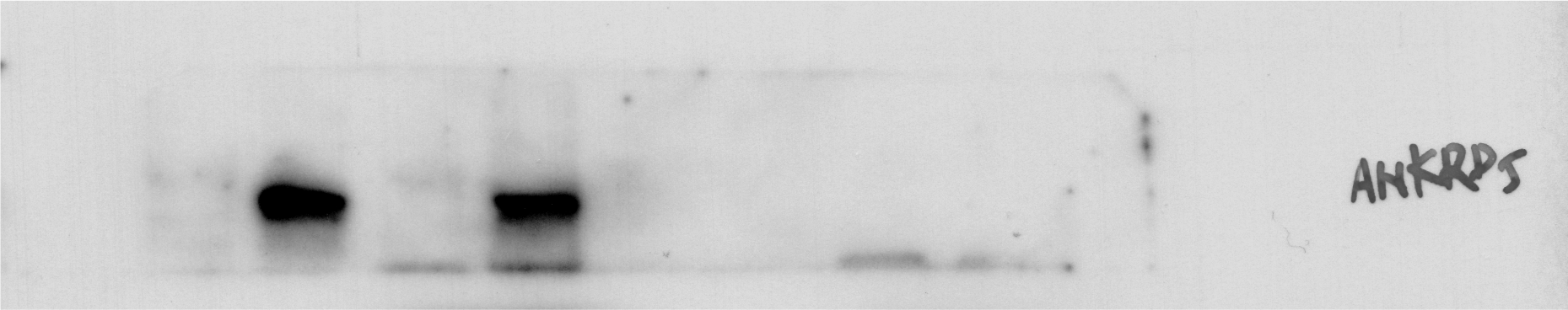

Supplement: Figure 5—figure supplement 1—source data 6. [file elife-105321-fig5-figsupp1-data6.zip › Figure 5-figure supplement 1_Source data 6/ANKEF1-Flag.tif]

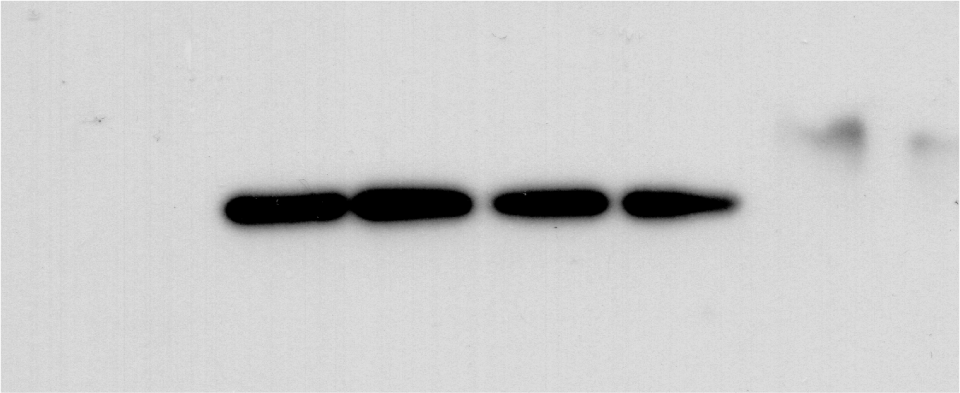

Supplement: Figure 5—figure supplement 1—source data 6. [file elife-105321-fig5-figsupp1-data6.zip › Figure 5-figure supplement 1_Source data 6/GAPDH.tif]

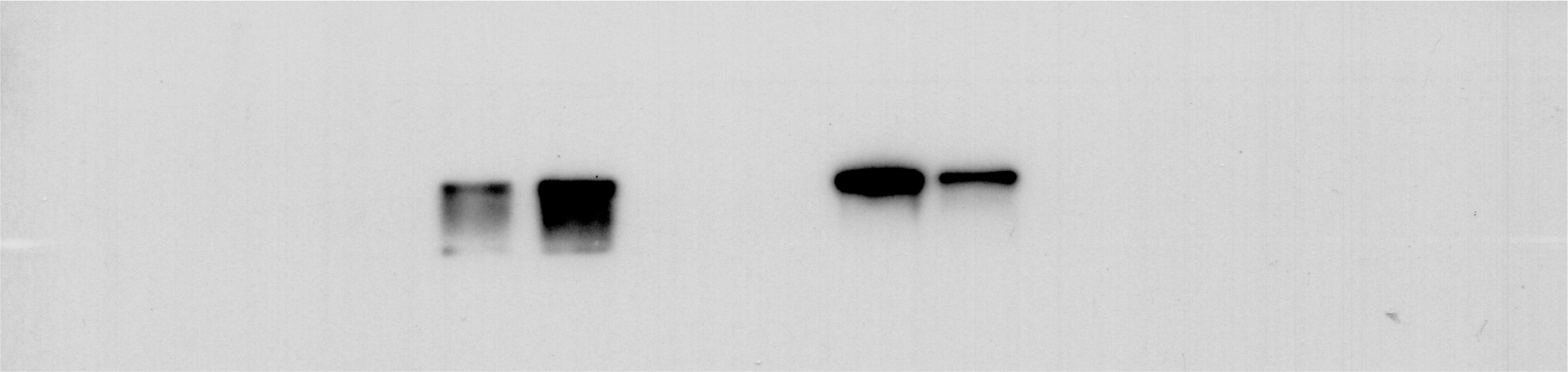

Supplement: Figure 5—figure supplement 1—source data 6. [file elife-105321-fig5-figsupp1-data6.zip › Figure 5-figure supplement 1_Source data 6/LRRC48-Myc.tif]

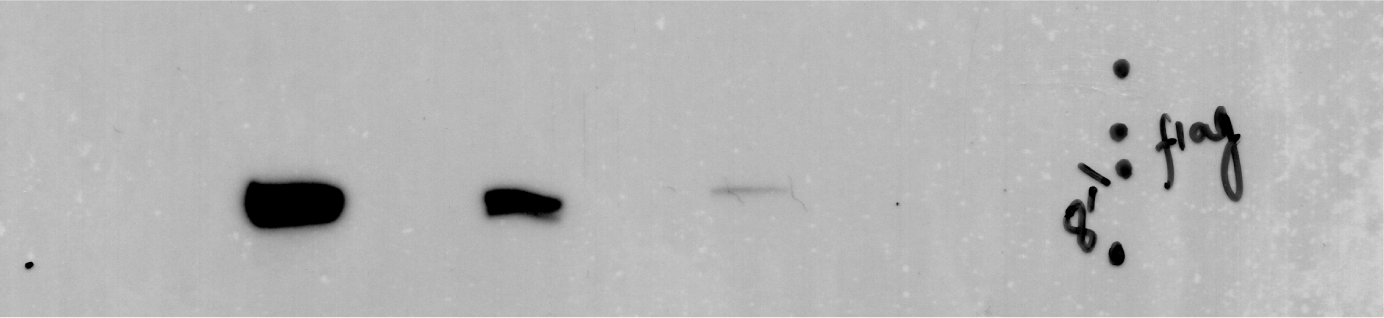

Supplement: Figure 5—figure supplement 1—source data 8. [file elife-105321-fig5-figsupp1-data8.zip › Figure 5-figure supplement 1_Source data 8/ANKEF1-Flag.tif]

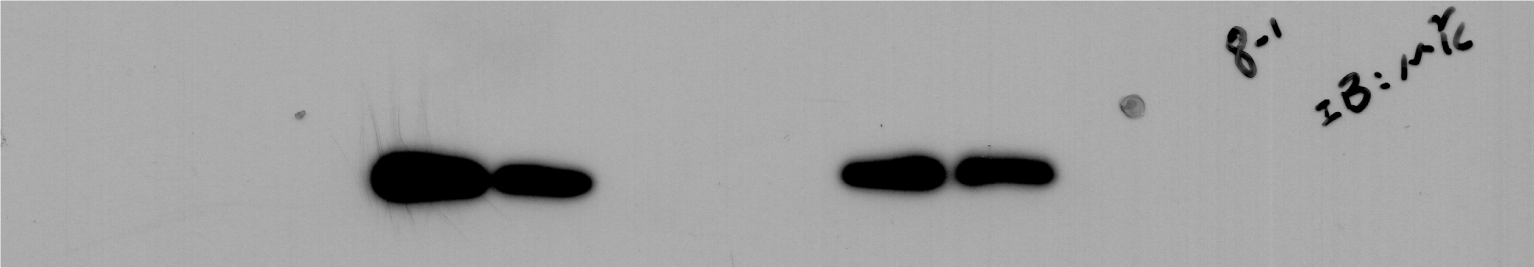

Supplement: Figure 5—figure supplement 1—source data 8. [file elife-105321-fig5-figsupp1-data8.zip › Figure 5-figure supplement 1_Source data 8/EFCAB2-Myc.tif]

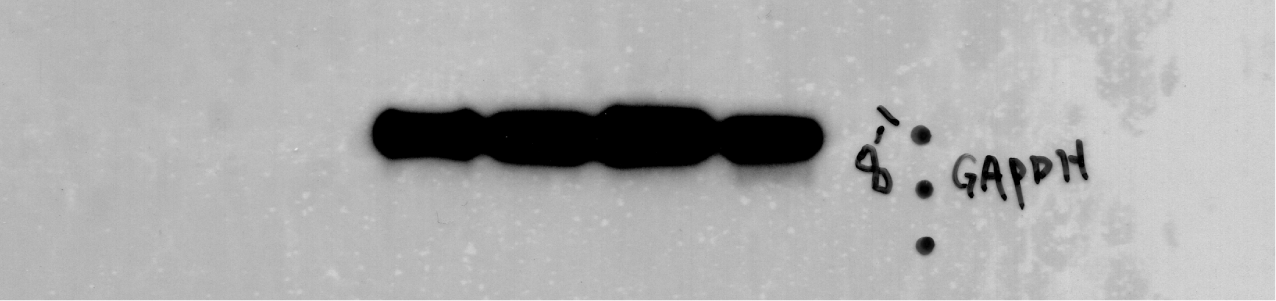

Supplement: Figure 5—figure supplement 1—source data 8. [file elife-105321-fig5-figsupp1-data8.zip › Figure 5-figure supplement 1_Source data 8/GAPDH.tif]

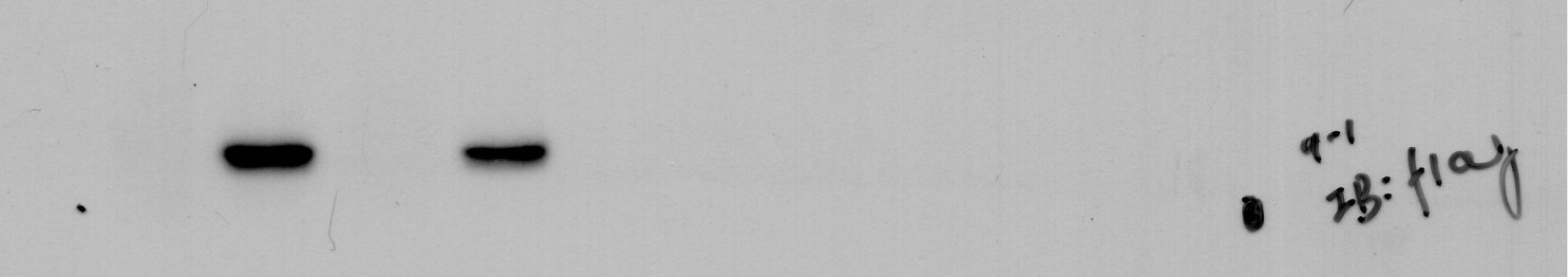

Supplement: Figure 5—figure supplement 1—source data 10. [file elife-105321-fig5-figsupp1-data10.zip › Figure 5-figure supplement 1_Source data 10/ANKEF1-Flag.tif]

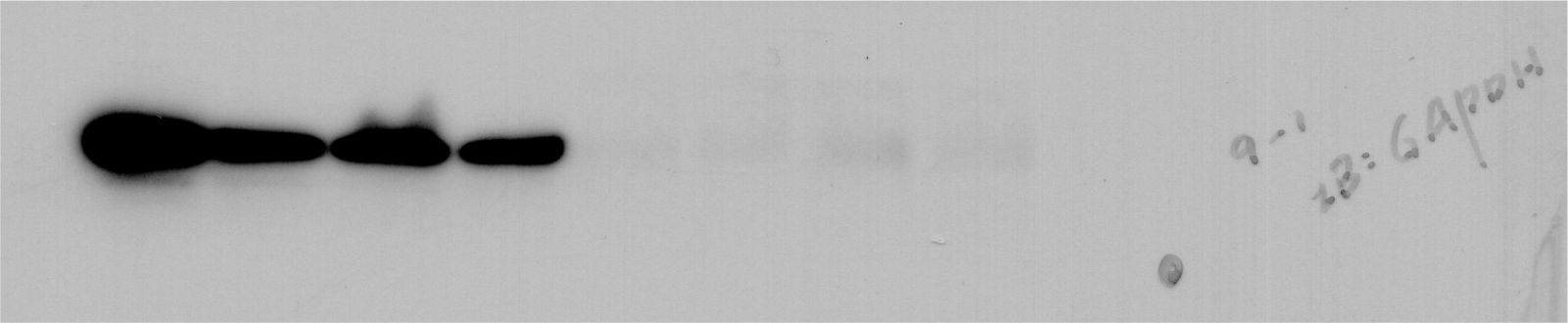

Supplement: Figure 5—figure supplement 1—source data 10. [file elife-105321-fig5-figsupp1-data10.zip › Figure 5-figure supplement 1_Source data 10/GAPDH.tif]

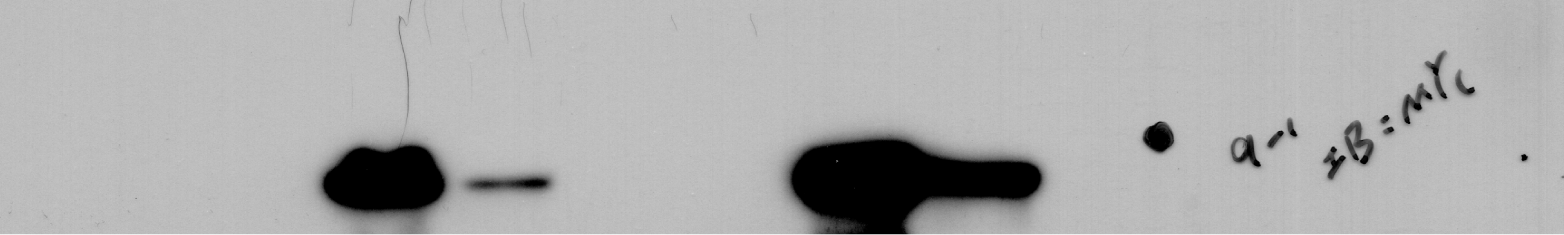

Supplement: Figure 5—figure supplement 1—source data 10. [file elife-105321-fig5-figsupp1-data10.zip › Figure 5-figure supplement 1_Source data 10/IQCG-Myc.tif]

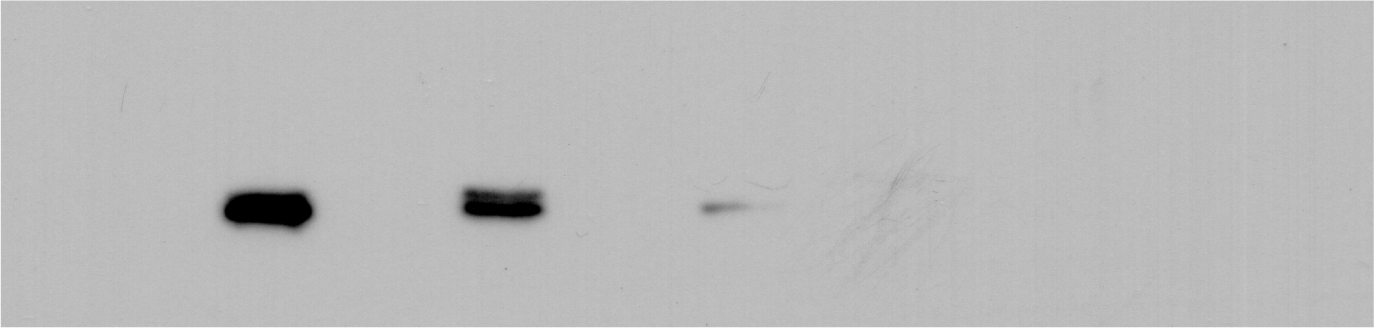

Supplement: Figure 5—figure supplement 1—source data 12. [file elife-105321-fig5-figsupp1-data12.zip › Figure 5-figure supplement 1_Source data 12/ANKEF1-Flag.tif]

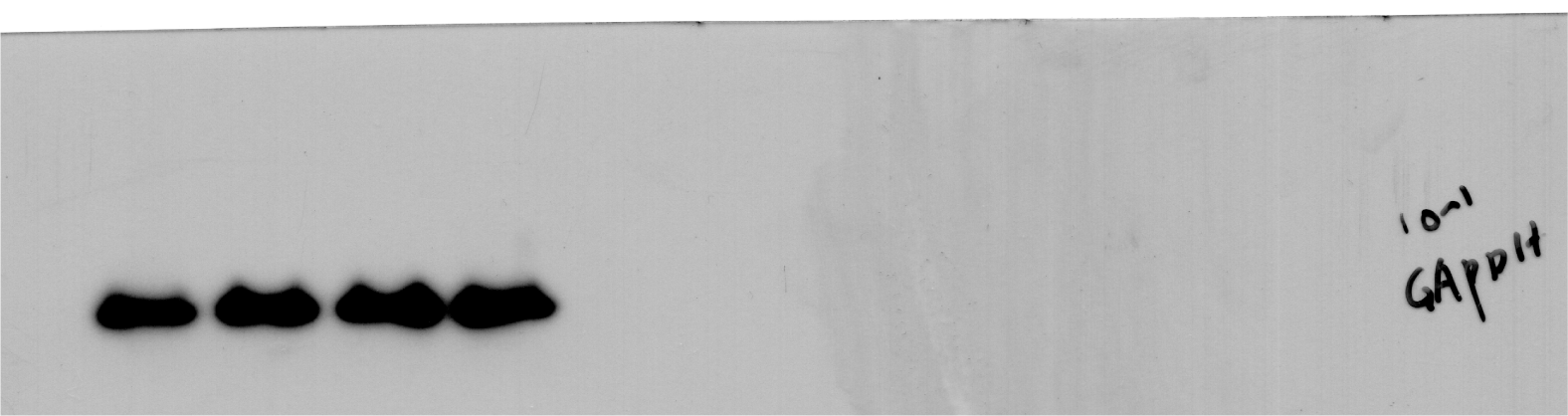

Supplement: Figure 5—figure supplement 1—source data 12. [file elife-105321-fig5-figsupp1-data12.zip › Figure 5-figure supplement 1_Source data 12/GAPDH.tif]

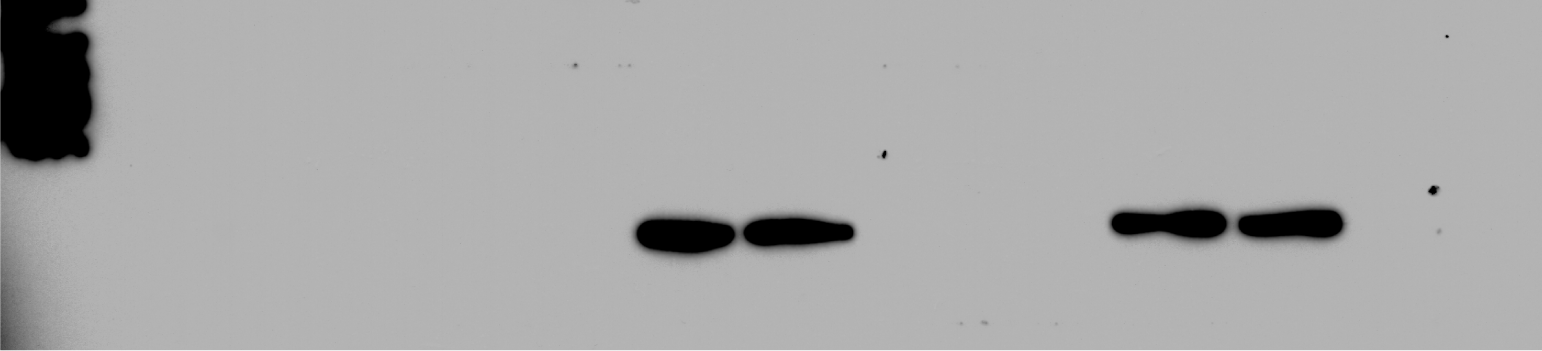

Supplement: Figure 5—figure supplement 1—source data 12. [file elife-105321-fig5-figsupp1-data12.zip › Figure 5-figure supplement 1_Source data 12/IQCD-Myc.tif]

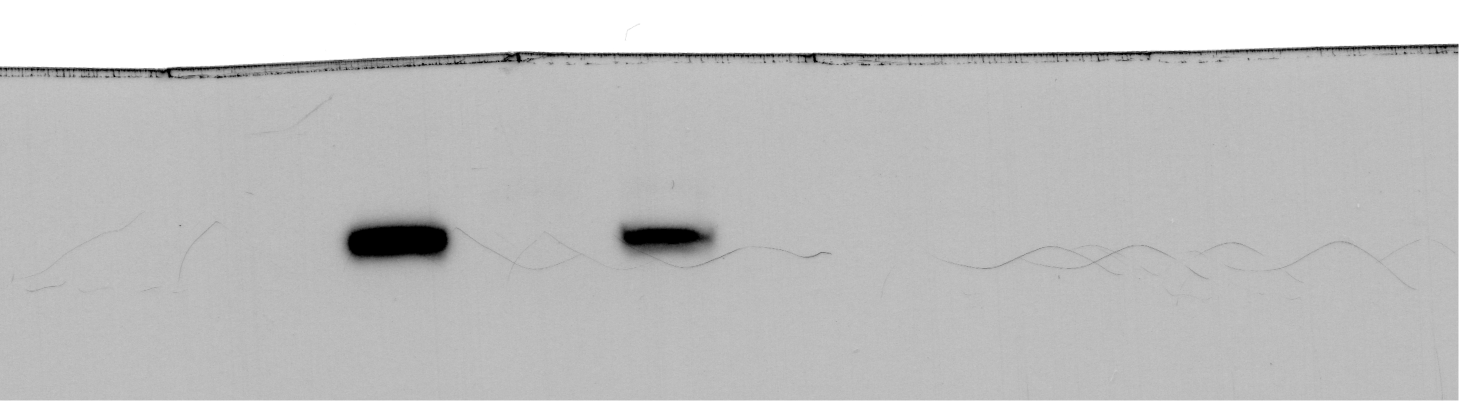

Supplement: Figure 5—figure supplement 1—source data 14. [file elife-105321-fig5-figsupp1-data14.zip › Figure 5-figure supplement 1_Source data 14/ANKEF1-Flag.tif]

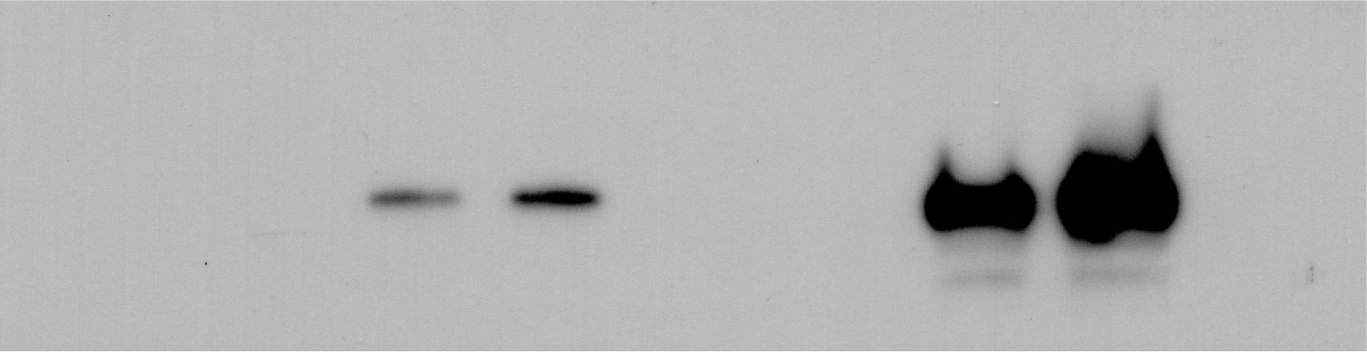

Supplement: Figure 5—figure supplement 1—source data 14. [file elife-105321-fig5-figsupp1-data14.zip › Figure 5-figure supplement 1_Source data 14/DRC7-Myc.tif]

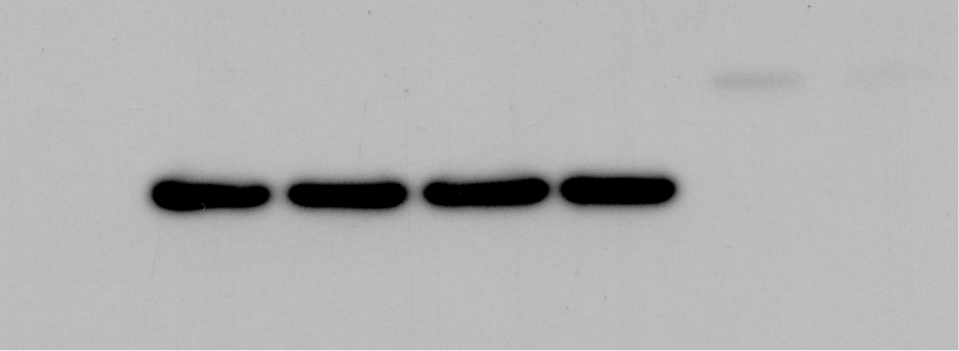

Supplement: Figure 5—figure supplement 1—source data 14. [file elife-105321-fig5-figsupp1-data14.zip › Figure 5-figure supplement 1_Source data 14/GAPDH.tif]

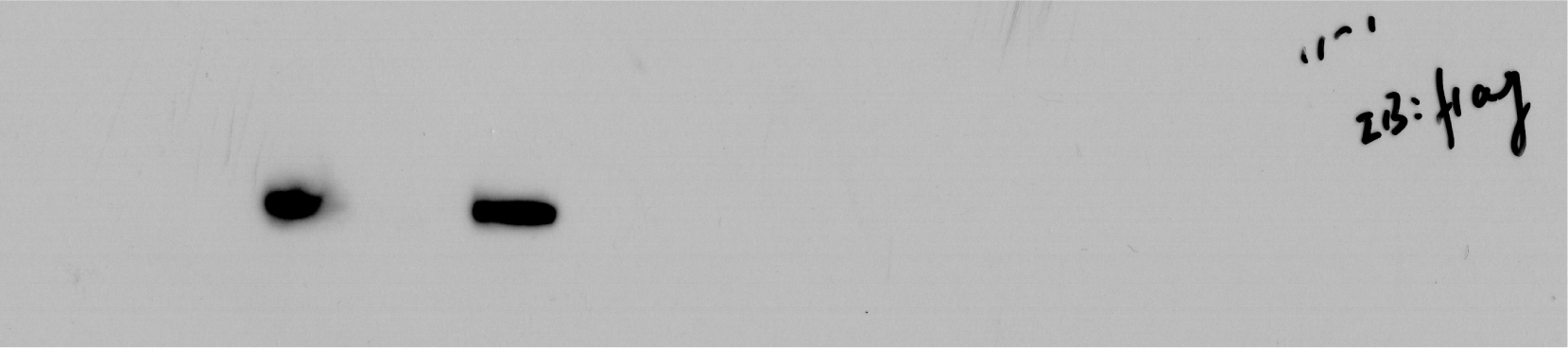

Supplement: Figure 5—figure supplement 1—source data 16. [file elife-105321-fig5-figsupp1-data16.zip › Figure 5-figure supplement 1_Source data 16/ANKEF1-Flag.tif]

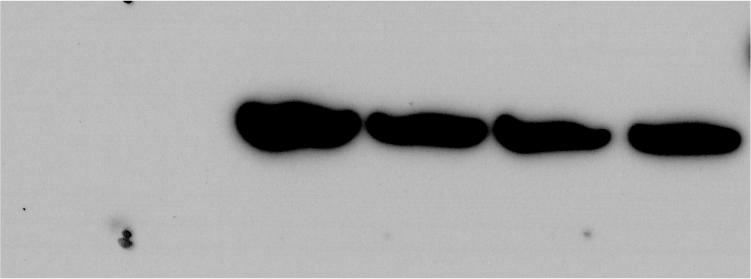

Supplement: Figure 5—figure supplement 1—source data 16. [file elife-105321-fig5-figsupp1-data16.zip › Figure 5-figure supplement 1_Source data 16/GAPDH.tif]

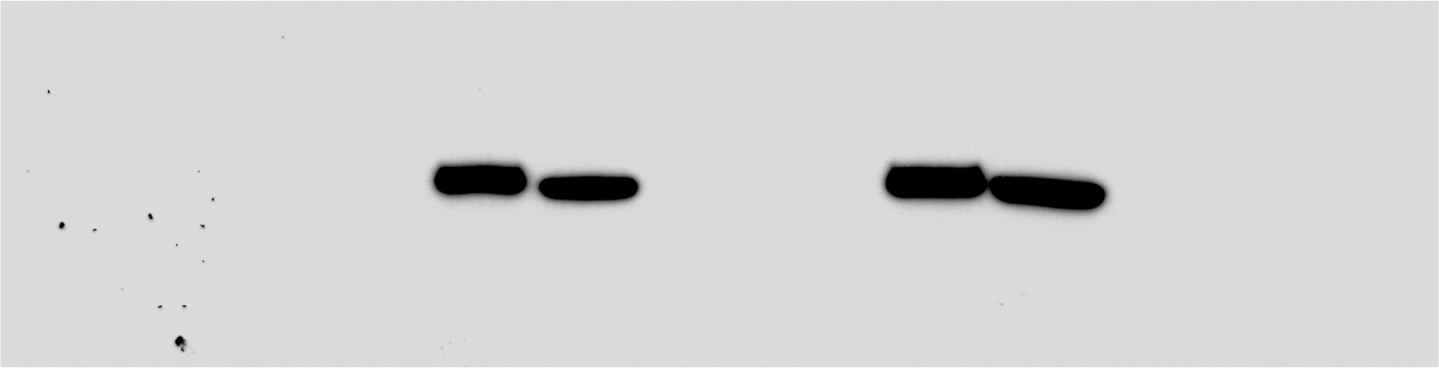

Supplement: Figure 5—figure supplement 1—source data 16. [file elife-105321-fig5-figsupp1-data16.zip › Figure 5-figure supplement 1_Source data 16/IQCA1-MYC.tif]
