# Supplementary material for: ANKEF1 is a key axonemal component essential for murine sperm motility and male fertility
Source: eLife. 2025 Dec 29;14:RP105321. doi: 10.7554/eLife.105321 (PMC12747526; doi:10.7554/eLife.105321)
Supplement: Figure 4—source data 3. [file elife-105321-fig4-data3.zip › Figure 4_Source data 3/Figure 4_Source Data 3.pdf]

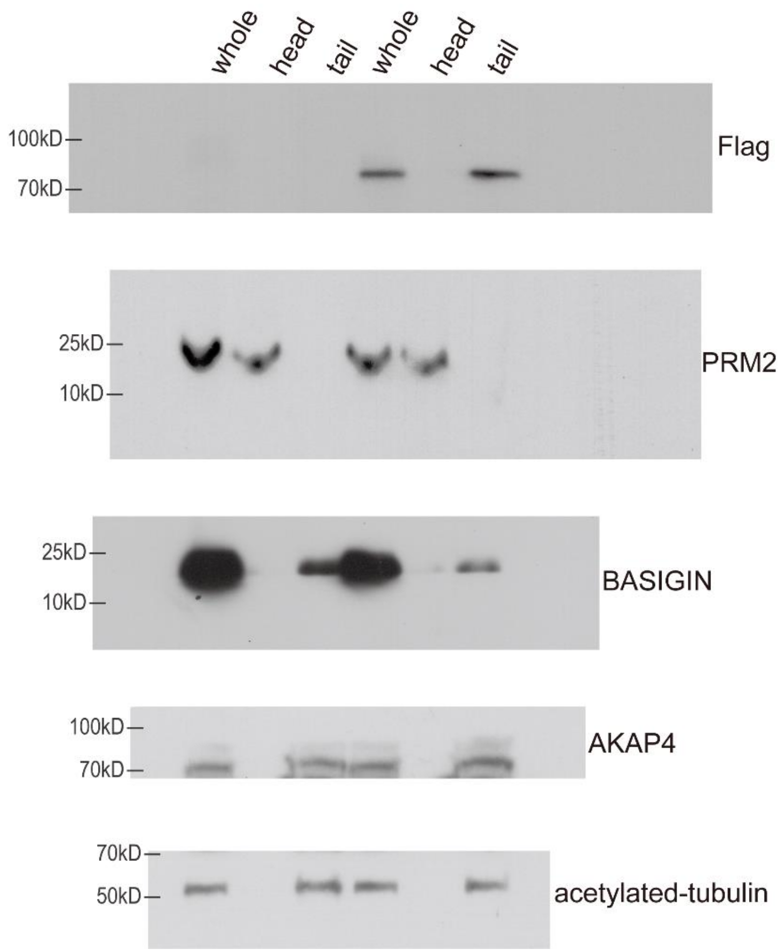

Figure 4, Source Data 3. Original, uncropped western blot membranes corresponding to Figure 4B. The membranes were sequentially probed with antibodies against: ANKEF1-Flag, PRM2 (sperm head marker), BASIGIN (membrane/cytosolic marker), AKAP4 (fibrous sheath marker), and acetylated tubulin (axonemal marker). For each blot, lanes correspond to: whole sperm, isolated sperm heads, and isolated sperm tails. Pre-stained protein molecular weight markers were used (See Supplementary File 2 for antibody details).
