# Supplementary material for: ANKEF1 is a key axonemal component essential for murine sperm motility and male fertility
Source: eLife. 2025 Dec 29;14:RP105321. doi: 10.7554/eLife.105321 (PMC12747526; doi:10.7554/eLife.105321)
Supplement: Figure 4—source data 5. [file elife-105321-fig4-data5.zip › Figure 4_Source data 5/Figure 4_Source Data 5.pdf]

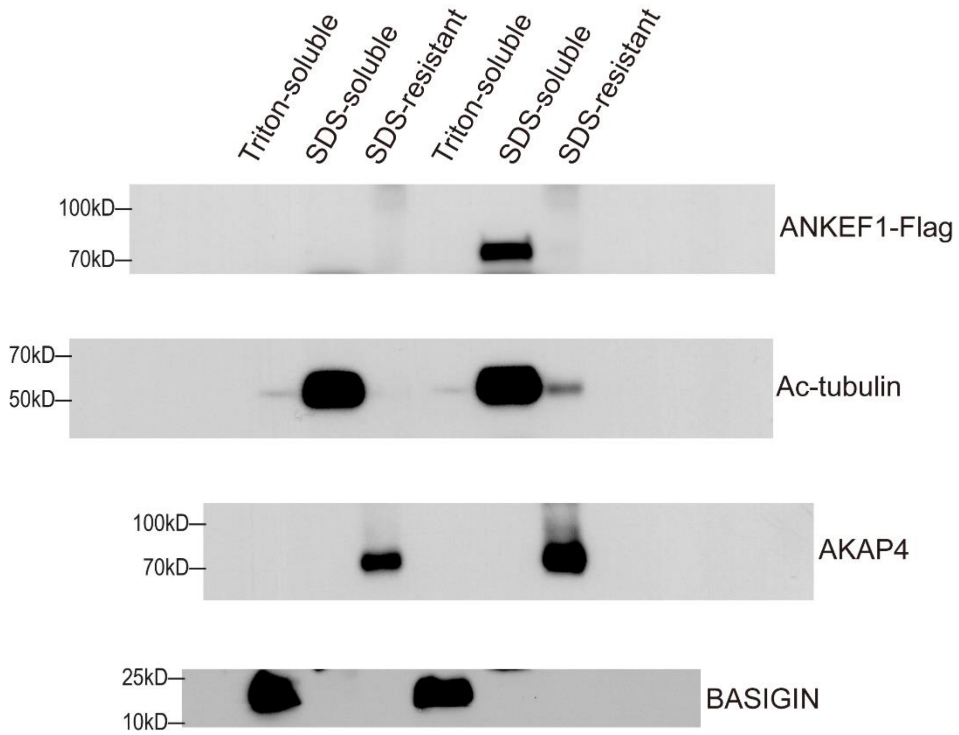

Figure 4, Source Data 5. Original, uncropped western blot membranes corresponding to Figure 4D. The membranes show the fractionation of sperm proteins using differential detergent solubilization. The blots were probed with antibodies against: ANKEF1-Flag, α-tubulin (loading control), AKAP4 (SDS-resistant fraction marker), and BASIGIN (Triton X-100-soluble fraction marker). For each blot, lanes correspond to: Triton X-100-soluble fraction, SDS-soluble fraction, and SDS-resistant fraction. Pre-stained protein molecular weight markers were used (See Supplementary File 2 for antibody details).
