# Supplementary material for: ANKEF1 is a key axonemal component essential for murine sperm motility and male fertility
Source: eLife. 2025 Dec 29;14:RP105321. doi: 10.7554/eLife.105321 (PMC12747526; doi:10.7554/eLife.105321)
Supplement: Figure 5—source data 3. [file elife-105321-fig5-data3.zip › Figure 5_Source Data 3/Figure 5_Source Data 3.pdf]

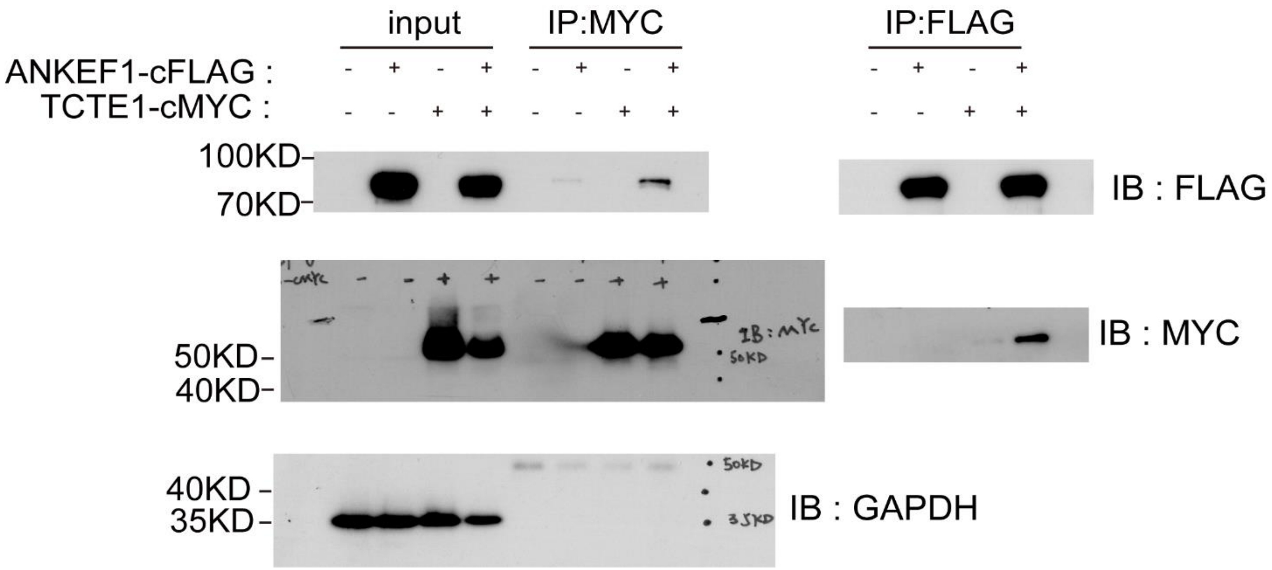

Figure 5, Source Data 3. Original, uncropped western blot membranes corresponding to Figure 5C. The membranes show co-immunoprecipitation (Co-IP) assays performed in HEK293T cells co-transfected with plasmids expressing ANKEF1-Flag and TCTE1-MYC. From top to bottom, the blots were probed with: anti-Flag antibody (detecting ANKEF1-Flag), anti-MYC antibody (detecting TCTE1-MYC), and anti-GAPDH antibody (loading control). Lanes correspond to: input lysates, proteins immunoprecipitated (IP) with anti-MYC antibody (IP: MYC), and proteins immunoprecipitated with anti-Flag antibody (IP: FLAG). "+" and "-" indicate the presence or absence of the respective transfected plasmid in each sample. Pre-stained protein molecular weight markers were used (See Supplementary File 2 for antibody details).
