# Supplementary material for: ANKEF1 is a key axonemal component essential for murine sperm motility and male fertility
Source: eLife. 2025 Dec 29;14:RP105321. doi: 10.7554/eLife.105321 (PMC12747526; doi:10.7554/eLife.105321)
Supplement: Figure 5—source data 7. [file elife-105321-fig5-data7.zip › Figure 5_Source Data 7/Figure 5_Source Data 7.pdf]

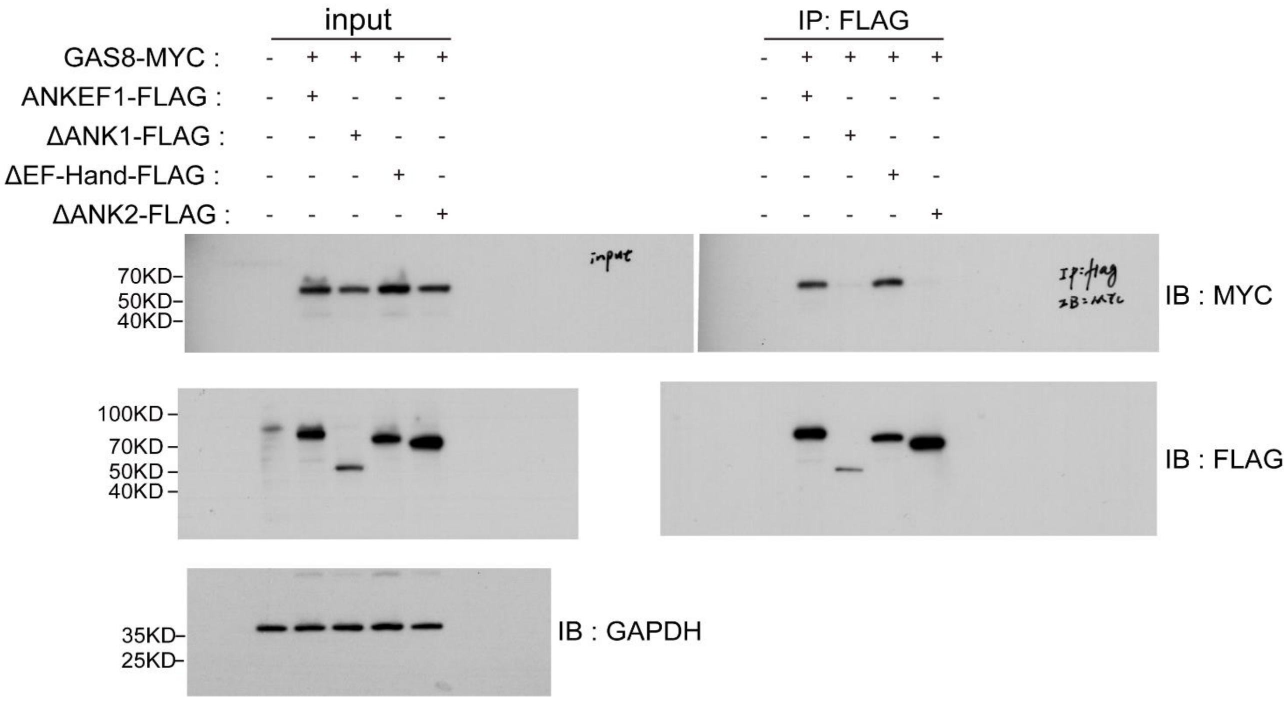

Figure 5, Source Data 7. Original, uncropped western blot membranes corresponding to Figure 5F. The membranes show co-immunoprecipitation (Co-IP) assays assessing the interaction between GAS8-MYC and various truncated forms of ANKEF1-Flag expressed in HEK293T cells. From top to bottom, the blots were probed with: anti-MYC antibody (detecting GAS8-MYC), anti-Flag antibody (detecting full-length or truncated ANKEF1-Flag proteins), and anti-GAPDH antibody (loading control). Lanes correspond to: input lysates (Input) and proteins immunoprecipitated with anti-Flag antibody (IP: FLAG). The schematic of each ANKEF1 truncation (ΔANK1, ΔEF-Hand, ΔANK2) is illustrated in Figure 5D. Pre-stained protein molecular weight markers were used (See Supplementary File 2 for antibody details).
