# Supplementary material for: ANKEF1 is a key axonemal component essential for murine sperm motility and male fertility
Source: eLife. 2025 Dec 29;14:RP105321. doi: 10.7554/eLife.105321 (PMC12747526; doi:10.7554/eLife.105321)
Supplement: Figure 5—source data 9. [file elife-105321-fig5-data9.zip › Figure 5_Source Data 9/Figure 5_Source Data 9.pdf]

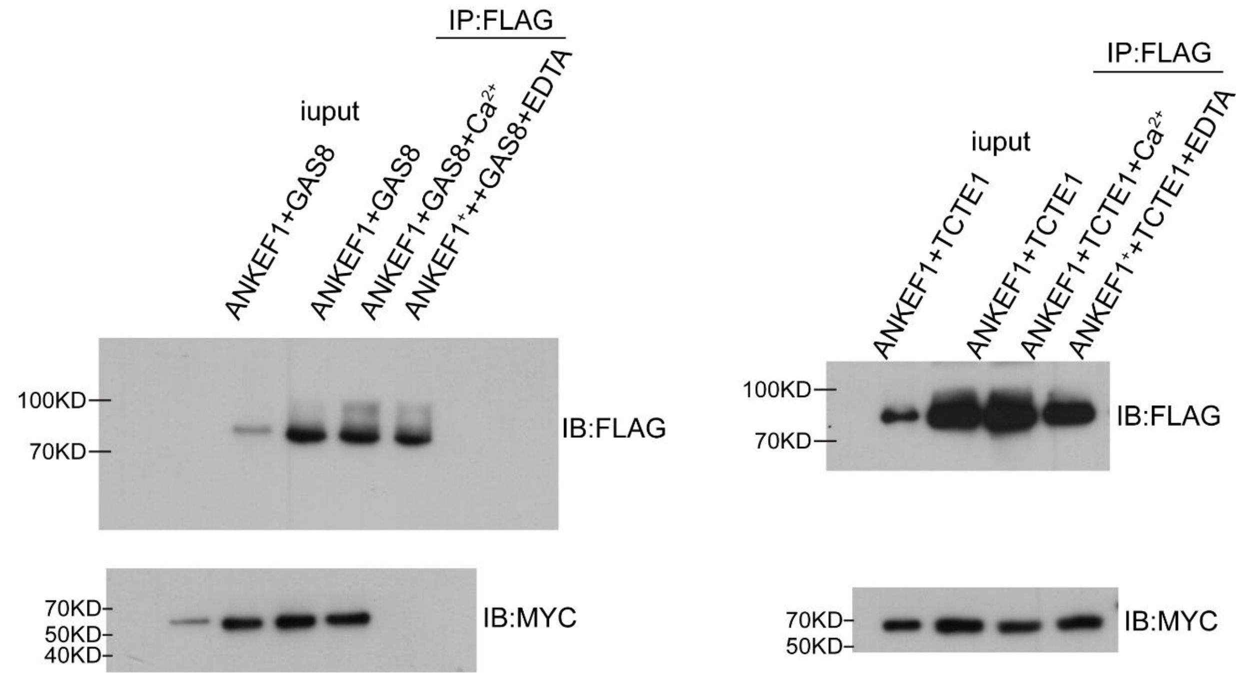

Figure 5, Source Data 9. Original, uncropped western blot membranes corresponding to Figure 5G. The membranes show co-immunoprecipitation (Co-IP) assays testing the effect of calcium ions (Ca^{2+}) and the chelator EDTA on the interaction between ANKEF1-Flag and GAS8-MYC (or TCTE1-MYC) expressed in HEK293T cells. From top to bottom, the blots were probed with: anti-Flag antibody (detecting ANKEF1-Flag), anti-MYC antibody (detecting GAS8-MYC or TCTE1-MYC), and anti-GAPDH antibody (loading control). Lanes correspond to: input lysates (Input) and proteins immunoprecipitated with anti-Flag antibody (IP: FLAG). Pre-stained protein molecular weight markers were used (See Supplementary File 2 for antibody details).
